# Metal and Metalloid Toxicity in Plants: An Overview on Molecular Aspects

**DOI:** 10.3390/plants10040635

**Published:** 2021-03-27

**Authors:** Paola I. Angulo-Bejarano, Jonathan Puente-Rivera, Rocío Cruz-Ortega

**Affiliations:** 1 Laboratorio de Alelopatía, Departamento de Ecología Funcional, Instituto de Ecología, Universidad Nacional Autónoma de México, UNAM, 275, Ciudad Universitaria D.F. Circuito Exterior s/n Anexo al Jardín Botánico Exterior, México City 04510, Mexico; pangulobe@tec.mx (P.I.A.-B.); jo_puenter@hotmail.com (J.P.-R.); 2 School of Engineering and Sciences, Centre of Bioengineering, Tecnologico de Monterrey, Queretaro 21620, Mexico

**Keywords:** abiotic stress, plant tolerance, metal toxicity

## Abstract

Worldwide, the effects of metal and metalloid toxicity are increasing, mainly due to anthropogenic causes. Soil contamination ranks among the most important factors, since it affects crop yield, and the metals/metalloids can enter the food chain and undergo biomagnification, having concomitant effects on human health and alterations to the environment. Plants have developed complex mechanisms to overcome these biotic and abiotic stresses during evolution. Metals and metalloids exert several effects on plants generated by elements such as Zn, Cu, Al, Pb, Cd, and As, among others. The main strategies involve hyperaccumulation, tolerance, exclusion, and chelation with organic molecules. Recent studies in the omics era have increased knowledge on the plant genome and transcriptome plasticity to defend against these stimuli. The aim of the present review is to summarize relevant findings on the mechanisms by which plants take up, accumulate, transport, tolerate, and respond to this metal/metalloid stress. We also address some of the potential applications of biotechnology to improve plant tolerance or increase accumulation.

## 1. Introduction

Metals and metalloid ions are a natural part of our planet and are present in the diverse layers that compose it. However, when their levels are found in high concentrations, toxicity for many of its life forms (microorganisms, plants, animals, and humans) can result [1,2]. In addition to their natural presence in the environment, human activity has largely contributed to their liberation from these natural sources, causing contamination of soils, rivers, oceans, and the atmosphere [3]. This has, in turn, impacted the food chains (bioaccumulation) and posing severe risks to human health through the pollution of arable lands and diverse ecosystems [3,4]. From an anthropocentric standpoint, the fact that many of these elements are now present in increasing concentrations represents a risk to global food security, along with various other forms of abiotic stress [4]. Recently, the distribution of this form of pollution in Europe, Africa, and China was revised [5]. In Latin American countries, the main sources for this form of environmental contamination are extensive agriculture, industrial, and mining activities [6]. The latter (mining) is considered one of the main sources of metal(oid) pollution, since many of the world’s metals and metalloids are extracted in Latin American countries [6]. In fact, in Mexico, mining has been a very profitable commodity since colonial times; the country is still the top producer of silver along with other metals of economic importance such as gold, copper, and lead, among others [7]. Important research has been done to analyze endemic, native, and invasive plants that can adapt to the conditions found in mine tailings, for example, the high concentration of copper found in ancient mining sites in Nacozari, Sonora, Mexico [8].

Heavy metals and metalloids can play important roles in plant development by participating in metabolic reactions and by acting as micronutrients (e.g., Fe, Co, Cu, Mn, Zn, and Mo) [2]. Nevertheless, when they exceed their threshold concentrations, their actions are considered toxic to plant development. The main characteristic used to classify heavy metals is density, which has been revised elsewhere [2,9]. In recent years, this term has been associated with the onset of a wide array of detrimental effects in plants. This is particularly true for elements such as arsenic (As), cadmium (Cd), lead (Pb), and chromium (Cr), among others [2]. Some other metallic elements, such as aluminum (Al), antimony (Sb), mercury (Hg), and nickel (Ni), among others, have also been studied to investigate their harmful effects in plant development when present above their threshold concentrations. For instance, aluminum toxicity in plants is related to the global increase in acidic soils (40% of the world’s arable land), since its most toxic forms (Al^3+^) are available under acidic pH values [10].

As stated previously, all metallic elements, whether they are related to plant development or not, have a threshold concentration beyond which deleterious effects and growth impairment are generated in plants [4]. In addition, the soil pH value is a very important aspect, since some elements are more bioavailable at pH ≤ 7 [10]. The harmful effect of an element in plants and other life forms relies on the capacity of such metallic ions to compete with normally occurring ions that are important cofactors or ligands for vital enzymes in primary and secondary metabolism [2]. Their interactions with sulfhydryl groups generates an imbalance in protein functions and an increase in the plant´s oxidative state [11]. In fact, they can displace important elements (e.g., Ca^2+^ and Mg^2+^) present in cell walls and membranes; for example, Al, Cu, Pb, and Zn bind more readily to the cell wall pectins than Ca [9].

Evolution has played a fundamental part in the adaptation processes of land plants, by enhancing the attributes necessary to thrive under various environments. This has occurred through multiple events, including speciation, duplication, and gene fixation among their genomes [12]. Hence, a plethora of complex mechanisms have developed in plant genomes to overcome abiotic stress. Plants also have the natural capability to thrive in metal- and metalloid-contaminated soils, which are a growing trend in many cultivable and arable lands worldwide [11]. Several research groups have gained interest in unveiling the mechanisms involved in the interactions among plants and metals, with the purpose of understanding plant evolution and also to take advantage of adaptation skills to utilize plants in phytoremediation strategies to alleviate the effects of increasing metal and metalloid concentrations in agronomically important soils around the globe [13].

In this sense, the plant–metal(oid) interaction at high concentration levels increases the oxidative state of plants, generating more reactive oxygen species (ROS) [9], and depending on the nature of these plants, the use of pre-existing coping mechanisms will be triggered or the expression of certain machinery to deal with the danger will be induced. Most of this damage is generated in the first contact zone for many potential hazardous metal(oids): the root. The root apical meristem (RAM), root cap, and root tip are the main sites for the first plant–metal(oid) interaction [14]. This, in turn, generates severe anatomical and physiological alterations to the root system, such as growth inhibition by compromising the functionality of meristematic cells present in the RAM, and the internalization of metal(oid) ions can also compromise elongation and proper root architecture formation [9,11,14]. Similar effects have been reported in lateral root primordia, where the presence of metallic ions generates cellular impairment and alterations in cellular hierarchy establishment in meristematic zones, leading to alterations in quiescent center (QC) formation [14]. Alongside this, root cortex tissues are compromised under conditions of metal and metalloid stress in plants, generating alterations in endodermal cells, deposition of suberin and lignin, cell wall thickening (exo and endodermal cells), formation of air spaces and alterations in intercellular spaces [11,14], and, finally, modifications to the root vasculature (central cylinder, parenchymatic cells in the pith), along with dark deposit formation (e.g., As (III) in *Glycine max* L.) [14,15]. Variations in the cell structure are not limited to the root system but protrude up to the stem tissues, disrupting cell division and enlargement in cortical cells and causing a loss of turgor in sclerenchyma cells in the vicinity of phloem cells, among other negative effects (e.g., after exposure to Cu, As, or Pb) [14]. Foliar tissues are the final frontier for metal(oid) uptake via the roots; thus, part of a plant´s plan is to avoid the entrance of such metallic elements into photosynthetic tissues by limiting their entrance or translocation to them [11]. However, completely avoiding this seems impossible in some plant systems, and therefore, negative effects appear, such as a reduction in leaf thickness, alterations to the epidermal cell structure and a reduction in intercellular spaces (mesophyll), an increase in callose deposition, and alterations to the stomatal structure, density, and aperture frequency (e.g., after exposure to Cd, As, and Mn) [14,16].

As will be discussed further, plants have evolved complex mechanisms to cope with metallic elements present above their threshold concentrations; thus, they are classified as hyperaccumulators (>1000 μg/g) and non-hyperaccumulators (<500 μg/g) [17]. General strategies for damage control include sequestration, exclusion, chelation, and speciation [5,11]. In general terms, it is accepted that important toxic metal elements are in contact with the root tip and can be internalized via symplast or apoplast, depending on their chemical nature [11]. Most of the plant´s constitutive transporters are part of the active transport system that functions with divalent cations (Ca^2+^ and Mg^2+^); therefore, many of the metal(oid) elements that have this chemical valence will find them suitable for entrance into the root tip [18]. Trivalent ions, such as Al^3+^, are not so abundant in nature but can be liberated due to human activity; thus, only one transporter has been related to Al^3+^ uptake in plants: NRAMP Aluminum Transporter 1 (NRAT1) [19]. Once the metallic ions cross the plant´s first barrier found in roots, they can be translocated using long distance transport through the phloem. In this regard, several important contributions have been thoroughly revised and discussed [11]. Briefly, the use of several important transporters has been described for hyperaccumulation and for tolerance mechanisms (in non-accumulator plants) and will be further discussed in this review.

Since the world’s population is predicted to increase to 9.1 billion by the year 2050 (FAO/WHO, 2021), and the increase in metal(oid) pollution is a trend that is affecting food security [4] and acting as an important cause for human illnesses, such as cancer, several strategies have been described for metal(oid) containment. In previous years, important reviews have gathered information and highlighted the importance of different strategies for metal pollution containment and phytoremediation; thus, we do not go into these in detail [20,21,22]. Briefly, phytoremediation-based strategies are considered green technologies that are cost effective and can be utilized as long-term solutions [20]. They include phytoextraction, phytovolatilization, phytostabilization, phytodegradation, rhizodegradation, and rhizofiltration [22]. Among these, phytoextraction is one of the most important for the removal of metals and metalloids in highly-contaminated sites, and the success of this technique depends on two main indicators: first, the metal concentration in roots/soils, which indicates the level of metal accumulation (also known as the bioconcentration factor (BCF)) and, second, the translocation factor, which represents the levels of metals that are effectively translocated from roots to shoots [23]. For this practice, plants must have important characteristics, such as high metal tolerance or accumulation, an elevated biomass, and the ability to grow rapidly [22]. To speed up this process, enhancer substances can be utilized such as chelator compounds [22].

Recently, the roles of biochar and compost have also gained importance for metal and metalloid containment in contaminated soils [24,25]. The use of biochar to reduce the toxic levels of heavy metals in such soils constitutes an ecofriendly and low-cost technology that also has advantages such as water retention and the immobilization of metal ions, such as Cu, Cr, Pb, Zn, and Mg, present at harmfully elevated concentrations [26]. In spinach (*Spinaccia oleracea*) and cilantro (*Coriandrum sativum*), the use of biochar reduced the concentrations of elements such as Cr, Zn, Cu, Pb, and Mn, and the bioaccumulation levels for these metal ions in these two plants [26]. Since biochar is drawing so much attention and has been revised recently, we do not go into more detail on this topic but encourage the reader to review related papers [24,25].

The aim of the present review is to gather relevant information concerning metal and metalloid toxicity and the mechanisms exerted by plants to either tolerate or hyperaccumulate these elements in their cells. Particular interest is placed on plant evolution regarding accumulation and tolerance traits, the different mechanisms of transport used, accumulation, transcriptome analysis, signaling pathways involved in the response to metals, changes in plant cells under heavy metal and metalloid stress conditions, and, finally, approaches to the biotechnological and ecological application of plants for possible phytoremediation purposes.

## 2. Plant Evolution for Hyperaccumulation and Tolerance

Changing environmental factors are most likely to exert profound effects on the evolution of organisms mainly by interfering with natural selection and through mutations, gene flow, and genetic drift processes [27,28]. When a population of individuals is exposed to certain contaminants or specific zones that could affect their survival or reproduction, the natural selection process will favor the survival of those that are capable of thriving in this environment [29], resulting in a completely evolved distinct population with new resistance mechanisms that contrasts with the genetic baggage of the sensitive population [30]. Whichever evolutionary process is responsible for the assimilation of metal ions in primitive life forms, the resulting organisms have to adapt to the formation of new protein structures with specific metal folds for metallic ions such as copper (Cu), zinc (Zn), and iron (Fe), among others, which allow them to maintain metal ion equilibrium and storage [28,31]. A determinant factor in the evolution of early living organisms is the existence of metal–organism interactions, because metal ions play crucial roles in structure, energy transport, and catalytic activities. Thus, metalloproteins (MTs) are considered to be among the initial proteins to have evolved and have a pivotal place in the establishment of primitive life milieus on Earth [32].

Among all organisms that have evolved metal tolerance and accumulation characteristics, plants are a fascinating example of adaptation to harsh and contaminated environments, and they have the ability to develop complex qualities through the natural selection process [9]. According to recent data, approximately 700 plant species out of the 300,000 vascular plants in existence are capable of undergoing metal hyperaccumulation [33]. They are represented by an extensive variety of taxonomic groups, exist in different geographic areas, and present a broad variety of morphological, physiological, and ecological characteristics [34]. Hyperaccumulating plants are normally endemic to soils that have important metal levels either naturally occurring (e.g., through the mineralization of parent rocks) or derived from human activities (e.g., mining and smelting) [35]. Plants with hyperaccumulation capacity are defined as those that are capable of growing in soils or environments where the concentration of a certain ion is considered high. Thus, the thresholds for certain elements have been set to the following values: Mn (10 mg/g), Zn (3 mg/g) As, Cr, Ni, and Pb (1 mg/g), and Cd, Se, and Tl (0.1 mg/g) [28,34].

Several adaptations to plants’ physiology that allow metal hypertolerance and hyperaccumulation are consequences of alterations to specific nodes in a very complex evolutionary network. Most of these modifications are associated with adjustments to root metal uptake, which is generally enhanced in hyperaccumulator and hypertolerant plants. In addition, metal transport through the symplast is more efficient, leading to an increment in the root to shoot the transport rate and involving a plethora of molecular or genetic mechanisms that work synergistically to efficiently distribute and store the metal(s) in shoot vacuoles [36]. Hyperaccumulative traits have appeared independently several times over the course of evolution [28]. However, the exact plant evolutionary mechanisms related to tolerance and hyperaccumulation traits are not very clear; thus, some authors have indicated that genes that confer tolerance do so at a cost to fitness, and they can only be manifested under certain combinations of conditions faced in the environment [35]. Hyperaccumulation is not considered a direct mechanism or a pleiotropic consequence of tolerance; if it was, the hyperaccumulative trait would be present in high levels amongst plants developing in highly contaminated soils [37]. 

While metal tolerance follows a positive relationship trend with the soil metal concentration, hyperaccumulation is not correlated with tolerance or the soil metal levels; conversely, the plant´s capacity to hyperaccumulate is negatively correlated with the metal levels found in native soils in every population, suggesting that hyperaccumulation and tolerance are not governed by identical evolutionary mechanisms [35]. In fact, plants with the strongest ability to concentrate metals are those from soils with the lowest metal contamination intensities. Hyperaccumulation and hypertolerance are independent traits at the genetic level; nevertheless, these traits are limited, since such a hyperaccumulating plant could poison itself if it did not possess a tolerance mechanism. In brief, five hypotheses for the possible adaptive benefits of metal hyperaccumulation have been stated: (1) machinery allowing increased tolerance, (2) metal-based allelopathy, (3) drought resistance, (4) an effective cation uptake mechanism, and (5) defense against herbivore and pathogen attack [35]. The ability to hyperaccumulate metals in plants constitutes a widespread divergence phenomenon with a high level of representation in at least three plant families: Asteraceae, Phyllanthaceae, and Brassicaceae [28,38]. The Brassicaceae family is one of the most studied. It includes the typical model plant *Arabidopsis thaliana* along with other members of this genus [39]. *A. thaliana* (a nonaccumulator species) and *A. halleri* (a hyperaccumulator and hypertolerant species) diverged around 3.5–5.8 million years (MY) ago. In addition, *A. halleri* and *A. lyrata* (a nontolerant species) diverged between 1.5 and 2 MY ago, and they share coding sequence identities of about 94 and 98%, respectively [40,41]. A distant relative of *A. thaliana* is *Thlaspi caerulescens* (or *Noccaea caerulescens*), which diverged about 20 MY ago and shares 88% identity [42,43]. However, this plant, along with *A. halleri*, has been utilized as a model to analyze the plant detoxification evolution process [36]. One example of convergent evolution among *A. halleri* and *T. caerulascens* is represented in HMA4, which is a Zn and Cd plasma membrane ATP-ase pump localized in the root pericycle and xylem parenchyma cells. The *HMA4* gene has an essential function in Zn/Cd hypertolerance and hyperaccumulation by mediating the root-to-shoot translocation of metals [44]. These two plant species diverged around 40 MY ago, and *HMA4* is overexpressed in both species due to gene tandem duplication and deregulated expression. The *HMA4* duplication event concurred with the speciation event amongst *A. halleri* and *A. lyrata* (that diverged much more recently than *A. halleri* and *T. caerulescens*), thus supporting the idea that the HMA4 locus overcame independent molecular modifications in these hyperaccumulator species. In addition, *HMA4* expression generates pleiotropic results as a gene response to Zn deficiency in root tissues, causing an increase in ZIP transporters that are specific and that may also transport Cd [45]. This information suggests that functional differentiation occurs to a certain extent among three *AhHMA4* copies when expressed in *A. thaliana*, stemming from differences in expression levels rather than in expression profiles [44]. In fact, all Cd hyperaccumulators that have been reported up to now also store high levels of Zn. However, not all Zn hyperaccumulators are Cd hyperaccumulators, which suggests that Cd adaptation to contaminated soils constitutes a more recent evolutionary event in plants. Therefore, the ability to store really low amounts of diverse elements in shoots gives *A. halleri* an evolutionary advantage when adapting to metalliferous soils [46].

## 3. Metal and Metalloid Toxicity in Plants

Metal and metalloid toxicity is generated by elements such as Cu, Fe, Mn, Zn, Ni, Co, and Al (some are considered micronutrients) and others, such as Cd, As, and Pb, that do not have biological relevance to plants and are associated with negative effects. Essential heavy metals are needed in minute quantities for vital functions (biochemical and physiological) [46,47]. When present above their threshold limits, all of these elements have different levels of toxicity in plants, which depend mainly on their concentrations and bioavailability in different soil types and have severe effects on the normal functioning of metabolic pathways by interfering or replacing pigments or enzymes and altering their original method of action [48,49]. The uptake and accumulation of heavy metals by plant roots occurs through an inter-related network of physiological and molecular mechanisms, including the binding of metals to extracellular exudates and cell wall components; compartmentalization of toxic metals from cytoplasm to the vacuoles; the formation of complexes of metals and amino acids, organic acids (OAs), MTs, or phytochelatin (PC); the production of heavy metal-induced antioxidative enzymes; and plant metabolism modification and repair and recovery of damaged cellular structures. These processes lead to changes to important physiological and biochemical processes, including adjustments to gene expression, protein variations, and alterations in the metabolite composition, all of which are responsible for the development of appropriate signals to activate defense and tolerance machinery in plants under conditions of heavy metal toxicity and detoxification mechanisms that may differ among plants and exposure to different metals or metalloids [50,51]. The symptoms of high metal concentrations in plants include chlorosis in young leaves and growth inhibition due to a decoupling of photosynthetic enzymes [52].

Every battle in this fight for survival has a starting point; in the case of metal toxicity, the interaction between an increasing soil metal concentration and root epidermis sensing and signaling seems to be the first step [53]. Once the plant has established a first defense line involving the exclusion and chelation of metal ions with OA, another strategy follows. In many cases, as previously mentioned, this relies on a metal concentration threshold [54]. This strategy involves the tolerance or entrance of the complex formed by metallic ions and OAs into the cell (metal–OA complex). As described in the next subchapters, the expression of transporters to liberate OAs and chelating compounds to neutralize these elements in the rhizosphere is one of the main strategies triggered in plants under conditions of metal/metalloid stress.

**Figure 1:**
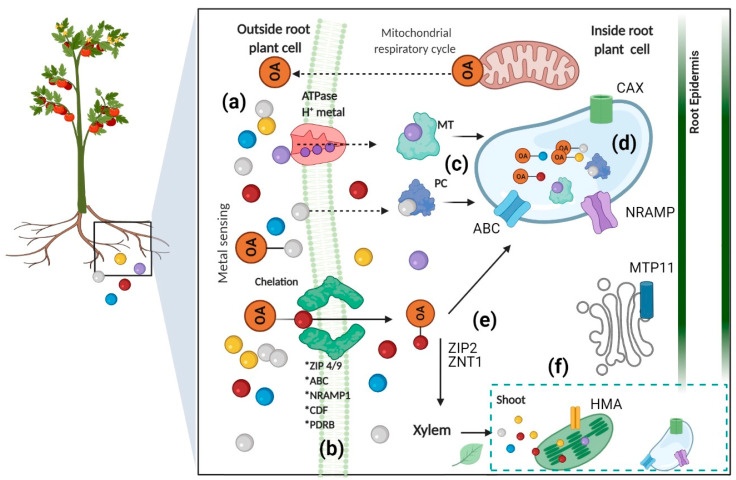
Schematic representation of the physiological and molecular processes of absorption/translocation of metals into plants. The uptake of heavy metals ((e.g., Pb, Cd, As, Zn, etc.) (colored circles)) occurs through the root cells, where the presence or high concentration of these metals triggers different signaling pathways inside the cell. The metal sensing signals initiate a defense response in plants such as the release of mitochondrial-derived OAs that form complexes with the metallic ions outside the root cell (**a**), or the introduction of metals and metal–OA complexes to cells through transporters (ABC-type, ZIPs, CDF, ATPase H^+^ metal, etc.) (**b**). In the cytosol, these metals form complexes with protein chelators (MTs and PCs) (**c**) that are then transported into vacuoles, also by metal transporters (ABC-type, NRAMP, CAX, and MTP), to accumulate there or to another organelle such as the Golgi (**d**). Heavy metals also can be translocated to the xylem by transporters (ZIP2 and ZNT1) and ultimately transported to the shoots (**e**), where they can also be introduced into the cell vacuoles, Golgi (MTP11), and chloroplasts (HMA) by transporters (**f**). Orange circles represent organic acids (OAs). MT, metallothionein, PC, phytochelatin.

These transporters include zinc–iron permease (ZIP), heavy metal transport ATPase (CPx- and P1B-ATPase), natural resistant associated macrophage protein (NRAMP), cation diffusion facilitator (CDF), and ATP-binding cassette (ABC) transporters, which are present at the plasma membrane and on the tonoplast membrane of cells. These are used to introduce the chelated metals/metalloids into the plant cells and to translocate them into accumulating organelles (cell wall and vacuoles) in plant tissues (leaves) [55,56]. ABC transporters are powerful transporters that drive the exchange of compounds across many different biological membranes, in most cases against electrochemical gradients using energy released from ATP hydrolysis [57]. They have been widely described as crucial elements in metal and metalloid toxicity in plants, which function as ATP-driven pumps, and their structure consists of two transmembranal domains (TMB), which are hydrophobic and form the membrane-spanning pores and two cytosolic domains. The latter domains are called “nucleotide binding domains (NBD)” or “nucleotide binding folds (NBF)” [58]. These types of transporters are well represented in different organisms and are highly conserved among species. Compounds that originate in the cytoplasm are pumped out of the cell by ABC transporters, and they also allow the entrance of compounds generated outside the plant cell and into intracellular organelles (endoplasmic reticulum, mitochondria, vacuole, and peroxisome, among others). The organisms with more complex functions require a greater number of transporters to exchange metabolites and information between cells and organs. In plants, the ABC and secondary active transporters have great relevance in the development, nutrition, abiotic stress defense, and overall interaction with the environment [59]. The *AtABCC1* and *AtABBC2* genes are important for metal access and sequestration into vacuoles, and their participation under conditions of metal stress has already been described for Cd (II), As, and Hg (II), among others. *AtABCC1* participates in the transport of xenobiotics by folate glutathione S-conjugates, whereas *AtABCC2* and *AtABCC3* carry out the transport of glutathione S-conjugates, mainly xenobiotics, among other compounds [60]. Further, some plants translocate the metal–OA complexes to the aerial parts of the plant by activating the expression of different metal transporters into the xylem to efficiently move these complexes to their final storage destination (Figure 1). We summarize some of the effects of metals in plants in Table 1. In the next subchapters, we cover some examples of the effects, both physiological and molecular, of metals and metalloids that are of biological relevance but are toxic in high concentrations (Zn and Cu), others that could have some beneficial effects in plants (Al), and finally, toxic elements (Pb, Cd, and As) with adverse impacts on plant functions. We emphasized the cellular response to counter the toxic effects and provide a molecular and biotechnological approach to the possible use of accumulative and hyperaccumulative plants. 

### 3.1. The Effects and Responses of Zinc (Zn) in Plants 

Zn is one of the main metals required for all live forms, it participates in many enzymatic activities, is part of some transcription factors, and is a cofactor for a great number of proteins [83]. The toxicity of Zn depends on its bioavailability, concentration, and exposure time, the plant genotype, and plant development steps. The main symptom of Zn poisoning in plants is the growth inhibition of young, greened leaves in seedlings, possibly due to reduced Fe^2+^ and Fe^3+^ intake, which may lead to cell death, growth changes caused by mitosis inhibition [84], and a reduction in biomass production as a result of photosynthetic machinery decoupling. This leads to a decrease in the total chlorophyll content and an increase in the levels of H_2_O_2_, malondialdehyde (MDA), and lipoxygenase activity [85]. However, in the presence of high Zn concentrations, plants are prone to upregulation of the expression of Zn transporters and upregulation of a complex network of Zn detoxification mechanisms to overcome this stress [86]. Nicotianamine serves as a chelator molecule for Zn, forming complexes with Zn (II), and interestingly, glutathione, PCs, and MTs can also bind to Zn [87,88]. Some other types of chelators, such as citrate and malate, are both relevant in the Zn detoxification process. In fact, FRD3 (ferric reductase defective) participates in the root to shoot translocation of zinc and in citrate exudation [89]. Furthermore, the zinc-regulated transporter (ZRT)/iron-regulated transporter (IRT) like proteins (ZIP) constitute a vast group of Zn transporters that are well characterized in plants. They participate in the primary uptake of Zn from the soil. Other important Zn transporters include AtMPT1 (metal tolerance protein 1), a tonoplast-associated protein that allegedly functions under conditions of basal Zn excess [90]. Its role is to sequester Zn and introduce it into vacuoles of young, dividing cells in shoots, generating a storage point in these tissues. Additionally, the expression of AtMPT increases under conditions of Zn, Mn, and Cd excess and Fe deficiency [66,86]. Excessive Zn levels are detoxified in part with the help of AtHMA3, and if this protein is absent, cytoplasmic levels of Zn cause an overload in chloroplasts [91]. *AtPCR2* is a gene that encodes the plant cadmium resistance (PCR) protein in *A. thaliana* and is related to Zn tolerance due to root–shoot translocation [67]. Yellow stripe (YS) and yellow stripe-like (YSL) proteins have also been associated with Zn transport in both monocot and dicotyledonous plants, respectively. The first were formerly described in maize as Fe(III)–phytosiderophores (transporting the metal from the rhizosphere to root cells), and they are capable of transporting Zn(II)–phytosiderophores [92]. *AtYSL1* and *AtYSL3* are genes found in *A. thaliana* that participate in Zn remobilization from the leaves to the seeds during senescence. Phytotoxical effects of Zn can be reverted by the addition of several compounds in plants, including proteins, phytohormones, and chemicals. The application of H_2_S gas to the roots of *Solanum nigrum* L. reduces Zn-induced growth inhibition by enhancing the expression of several antioxidative enzyme genes and MT while decreasing Zn accumulation [93]. The exogenous foliar application of 24-epibrassinolide (EBL) and 28-homobrassinolide (HBL) in *Raphanus sativus* L. was shown to enhance the levels of ascorbate (ASA), glutathione (GSH), and proline to confer resistance to radish plants against Zn^2+^ stress with positive effects on the restoration of photosynthetic pigments [94]. Additionally, in Zn-stressed seedlings of *Carthamus tinctorius* L., oxidative damage can be reverted by the exogenous application of melatonin, GSH, or a combination of both with increments in ascorbate ASA, GSH, and PC [85].

### 3.2. Induction of ROS by Copper (Cu) and the Response in Plants 

Plants require low quantities of toxic metal to act as parts of different metalloproteins and as cofactors for many different metabolic pathways involved in plant development and growth. In this sense, Cu is an essential element for the proper activity of plastocyanin, cytochrome oxidase, superoxide dismutase, and ascorbate oxidase as part of the antioxidant defense [95]. Accordingly, the Cu content in leaves is estimated to be 10 μg g^−1^ dry weight; higher concentrations are considered toxic in many plant systems [96]. Among the alterations triggered by Cu in plants, we can highlight negative effects on several vital cellular processes such as photosynthesis, electron transport, cell wall metabolism and senescence, increases in proline, chlorophyll and H_2_O contents, and effects on P uptake in roots via transporters [97,98]. Cu also elicits an imbalance in the overall redox state of cells by ROS production and lipid peroxidation. Furthermore, ROS generated by Cu are capable of reacting with thiol groups through Fenton chemistry, causing severe changes in protein structure, altering the proteins’ functions, with subsequent effects on metabolic pathways, and causing damage to DNA and other biomolecules [99], thereby changing the antioxidant machinery of the cell [100]. The oxidative stress caused by Cu also increases the H_2_O_2_ level, causes lipid membrane damage, leads to the accumulation of Cu in roots and shoots, and results in developmental inhibition (biomass reduction) and ion liberation from cells, which causes damage to proteins and nucleic acids [75]. To avoid the generation of ROS, intracellular Cu is chelated and delivered to its partner proteins by specific chaperones such as CCH and ATX1 (for Antioxidant Protein 1), which mediate the transfer of Cu to Cu-transporting ATPases [101]. Sodium nitroprusside (SNP) is a NO donor that could also help to reduce the metal toxicity elicited by Cu, Cd, Al, and As in plants [102,103]. However, plants also possess detoxifying mechanisms for cytotoxic compounds formed by reactions with Cu. Nitric oxide (NO) is involved in signaling pathways that, in many cases, lead to the activation of genes that participate in redox and defense activities, which help the plant to establish defense responses to overcome stress [104]. The application of a combination of exogenous GSH and SNP in Cu-treated *O. sativa* seedlings was found to ameliorate the effects of Cu toxicity through a reduction in levels of both lipid peroxidation and ROS-related enzymes [78]. Different cotton genotypes pretreated with salicylic acid (SA) and ascorbic acid (AsA, vitamin C) were associated with a decrease in oxidative stress in Cu-treated plants and a lower level of toxicity under these conditions. SA participates in plant signalization with a marked effect on antioxidant enzymes, inhibiting their translocation [105], whereas AsA regulates cellular processes like photosynthesis, cell expansion, root elongation, and transmembrane electron transport and causes marked exclusion and a decrease in Cu uptake [76].

### 3.3. Aluminium Response Mechanisms: Exclusion and Tolerance in Plants

Aluminum (Al) is the third most abundant element overall, after silicon and oxygen. It is found in food, air, soil, and water. Under acidic conditions, Al is solubilized into [Al(H_2_O)_6_]^3+^ in which the Al^3+^ cation is highly toxic to many plant species and causes serious effects on plant growth [71,74]. Al toxicity is triggered by acidic soils with a low pH (pH < 5.0) [106] and causes direct inhibition of root elongation and interferes with plant nutrient uptake; hence, it is considered a primary limiting factor of plant growth and development in acidic soils [98,107]. Since this element is extensively present in the Earth´s crust and can pose mild to highly toxic effects in plants, recent reviews have already addressed the main aspects regarding the physiological, metabolic, and molecular regulation of its many effects in plant homeostasis. Some examples are shown in [108,109].

Therefore, this section is limited to a general description of the main aspects of Al tolerance and exclusion machinery in plants. As stated previously, when soluble, this element is converted to toxic forms that induce severe damage in plant roots, especially in non-tolerant ones. For instance, Al can induce stress in rice roots, leading to severe anatomical changes that include modifications to the length and diameter of xylem vessels, reductions in size of the metaxylem vessels and vascular bundles, and stomatal closure, which limits photosynthetic activity [110]. The increase and accumulation of ROS levels in plants leads to oxidative stress, reducing the levels of important antioxidant enzymes such as catalase (CAT), superoxide dismutase (SOD), glutathione S-transferase (GST), and peroxidase. Additionally, if this oxidative state is prolonged, chromosomal aberrations and cell membrane peroxidation can occur, inducing negative effects on photosynthetic activity, damaging the cell structure, decreasing stomatal conductance, and, finally, leading to programmed cell death or apoptosis [111].

Exclusion and tolerance are the two main strategies developed by plants during the course of evolution to overcome Al toxicity, whereby the plant cell prevents the entrance of Al into the roots (apoplast and symplast), while in the case of tolerance, Al is transported inside the cell where it can be sequestered into organelles and detoxified [71,90]. This process includes the activation of various transporters such as the Al-activated malate transporter (ALMT) family (anion transport) and the multidrug and toxic compound extrusion (MATE) family (OA/H^+^ antiport transport), which aid the cell in the liberation of OAs (citrate, malate, oxalate, phenols, and polypeptides through roots into the soil acting as ligands to chelate Al ions, forming stable complexes) into the rhizosphere to avoid the entrance of Al into the plant cell [112,113,114]. Once inside the cell, the metal–OA complexes can be accumulated in vacuoles with the intervention of carrier molecules such as phytochelatins (PC) and metallothionein (MTs). Al translocation and accumulation are possible in a very narrow group of plants such as common tea (*Camellia sinensis*), hydrangea (*Hydrangea* L), buckwheat (*Fagopyrum esculentum*), and *Melastoma malabatricum*. These plants have the capability to accumulate above Al concentrations of 1000–3000 mg/kg or higher [71].

A different way of exerting Al tolerance is through mediation by other protein complexes formed by ABC transporters that are sensitive to Al rhizotoxicity: STAR1 and STAR2. They also participate in UDP–glucose efflux into the cell wall, modifying its composition and decreasing the capacity of Al to bind to the cell wall [19]. In fact, studies on non-model plant systems, such as *Andropogon virginicus* L., have revealed that ABCG type transporters are colocalized in roots and leaves with Al absorption areas. Specifically, the expression of such proteins has been observed in cell membranes, root endo- and exodermis, and the vascular bundle sheath and epidermal cell layers, thus indicating their possible role in Al-accumulation in plants [109].

Likewise, our research group reported a *FeALS3* sequence that is involved in Al tolerance in buckwheat [73]. Gene expression was found to be constitutive and increased when the Al concentration was augmented. Interestingly, increasing levels of abscisic acid (ABA) were correlated with an increase in *FeALS3* expression, suggesting a role for this phytohormone in Al tolerance in *F. esculentum* [73]. Recently, it has been demonstrated that, after the first 48 h of exposure to the threshold Al^3+^ concentration (50 µM) in buckwheat, the levels of important antioxidant enzymes increase along with the ABA levels, implying that they might participate in root recovery mechanisms [115]. Other elements and phytochemical compounds play roles in Al tolerance mechanisms. For instance, elements such as magnesium (Mg) can act by displacing/competing with Al for binding sites within root cells; or by increasing citrate exudation, influencing the upregulation of MATE-like genes [19].

In *Camellia sinensis,* a recent study indicated that phytochemical compounds such as epigallocatechin gallate and polymeric proanthocyanidins were detected colocalized in the same tissues with high Al accumulation, roots, and old leaves. These polyphenols can form complexes with Al, demonstrating their roles in the high accumulation capacity exhibited by tea plants [116]. The implication of phenolic substances has already been described in other plant systems such as *Eucalyptus camaldulensis* [117].

### 3.4. Toxic Effects of Lead (Pb) in Plants and Their Response

Around the globe, there has been a trend for a continuous intensification in the liberation of pollutants such as Pb, and many ecosystems have been affected by this toxic metal that is hard to degrade [118]. This represents a serious problem for different plant species, since it is not part of any metabolic pathway, and low concentrations are considered toxic [119]. In general, most of it is translocated to the shoots after its absorption into the roots using passive mechanisms that rely on H+/ATPase systems; thus, most of these metal ions are first translocated via apoplast through the endodermis (which acts as a natural barrier in deeper tissues, such as the stele) and then transported via symplast to vascular tissues [79,120].

Lead affects many physiological characteristics, generating growth impairment, with subsequent effects on the proper development of roots (swelling and stunted growth), seeds, and seedlings, increasing the presence of necrotic lesions, causing leaf chlorosis, reducing the seed germination rate, decreasing the root/stem biomass, and altering the water status, mineral nutrition, and enzymatic activity [79]. Perhaps one of the most profound effects is observed in the overall plant photosynthetic machinery [121], where the electron transport processes are disrupted, causing a four- to five-fold decrease in the energy transformation efficiency of photosystem II (e.g., as registered in wheat) [56,121,122].

Plants in contact with Pb respond in different ways depending on the ion concentration, level of exposure, and the plant’s developmental stage; however, when Pb levels are low, the plant can adapt to the conditions [123]. Among the Pb tolerance mechanisms described in plants, we can consider (1) eliminating Pb through cell wall pumps; (2) uptake reduction; (3) chelation with thiol compounds in the cytosol with the aid of PCs and MTs; (4) sequestration and inactivation in organelles (vacuoles); and (5) detoxification of Pb-induced ROS generation [80,81,124]. On the other hand, prominent levels of this element cause stimulate ROS production, and plants lack the ability to overcome this event [80]. This also produces an upregulation of genes that code for enzymes that contribute to this process, such as glutathione reductase, GST, ascorbate peroxidase, and SOD [81]. Pb also triggers modifications to leaves in sensitive plants, such as thylakoidal membrane disorganization, alterations to chloroplast morphology, nuclear membrane disruption, and the formation of electrodense deposits [124]. In resistant plants, interactions with Pb were shown to modify the structure of mitochondria and cause nuclear membrane disruption in root cells [82].

### 3.5. Effects, Response, and Cadmium (Cd) Tolerance in Plants

Cadmium is highly soluble in water with a long half-life; therefore, it accumulates in soil and is potentially toxic to plants. Thus, when it is absorbed by commercial crops, it is a risk to plants used for human or animal consumption [125]. Higher Cd concentrations can be toxic to plants, especially in roots, where it can be absorbed into the rhizodermis and root cortex, either through apoplastic or symplastic pathways, and then through the plasma membrane of the endodermis, before entering the stele for long-distance transport. This metal can be readily absorbed and sequestered into root vacuoles or transported into the xylem to be distributed along the entire plant; thus, Cd accumulation is greater in roots compared with the plant’s aerial parts [126,127]. 

As with many other toxic metals/metalloids, high Cd concentrations in plants cause toxicity at the physiological, morphological, and molecular levels [128] and can affect important cellular mechanisms, causing an overall decrease in plant growth, changes in nutrient uptake and bioavailability, and changes in the photosynthetic machinery [69,70,129,130].

Reductions of root length and growth are the main plant physiological responses to Cd stress [131]. In *A. thaliana*, Cd also induces the expression of transport genes such as *AtABCC3*, which, when upregulated, can rescue the phenotype in an *atabcc1 atabcc2* double knockout mutant under conditions of Cd toxicity. In fact, the highest *AtABCC3* levels were observed when Cd concentrations were also high, indicating that this gene is modulated by an increase in the Cd concentration [132].Further, Cd can compete with essential metallic elements such as Zn, Ca, and Fe for transport, inducing mineral deficiencies, interrupting the activity of important enzymes, and thereby, inhibiting the flow of electrons which can react with oxygen and deplete the pool of reduced glutathione due to its elevated affinity to thiol groups [133,134]. In *A. thaliana* and *A. halleri*, HMA4 and NAS2 (Nicotianamine Synthase 2) participate in the translocation of both Cd and Zn from the roots to the shoots. The former codes for a heavy metal ATPase, while the latter helps to increase the level of nicotinic acid, which plays an important role in metal/metalloid homeostasis [135]. Cd also induces adaptative response (AR) through metabolic blockers of protein kinase cascades, DNA repair, oxidative stress, and de novo translation [136]. The general Cd tolerance mechanisms involve (1) reducing its absorption and transport from roots to shoots; (2) compartmentation in the cell wall and chelation in root cell vacuoles; (3) enhancing the concentration of antioxidants and antioxidation enzymatic activity; and (4) upregulating the synthesis of PC and changing the expression of heavy metal transporter genes [137].

### 3.6. Arsenic (As) Uptake, Response, and Detoxification by Plants 

Arsenic is a nonessential metal compound that is also one of the most dangerous elements found as a product of human pollutant activities; it is indisputably top of the list when considering elements with negative effects on plants. This metal is present as three main species in soils and water: arsenite (As (III)) and arsenate (As (V)) (the most abundant form in soil) and methylated forms (monomethylarsinic acid (MMA) and dimethylarsinic acid (DMA)). The uptake mechanism differs for each ionic form [138,139,140]. As with other metals, plants utilize existing transporters for toxic metal uptake. For instance, As (V) enters the plant by utilizing phosphate transporters of the PHS family (PHT1 type, competing for Pi), which allow As uptake/translocation or accumulation, triggering changes inside the cell [138,141]. Conversely, arsenite can enter plant cells via aquaporins belonging to the nodulin 26 like intrinsic protein family (NIPs) [142]; furthermore, transcription factors of the WRKY family are also involved in its uptake [143]. The two mechanisms of arsenic entrance in the cell lead to alterations in plant developmental processes, and both forms of this element cause an oxidative burst in the form of ROS [144]. Arsenic uptake takes place through the root system (roots and tubers accumulate As in large amounts) [138,141]. However, some reports indicate that not all As is translocated to the shoots, and it is not accumulated in high amounts in edible plant parts [145].

Arsenic detoxification processes and tolerance mechanisms require the exclusion of the metal ions from cells and their compartmentalization into organelles, which involves proteins with metal affinity such as PCs and accumulation vacuoles [63,146]. To limit ROS accumulation, plants require actions by a series of enzymatic antioxidants—SOD, POD, CAT, APX, GR, and GST—and non-enzymatic antioxidants—ascorbic acid, proline, and cysteine [141]. Nitric oxide (NO) and silicon (Si) can mitigate As toxicity [147], and the exogenous application of NO protects *Vicia faba* plants against the adverse effects of As [143]. Salicylic acid also induces NO production, which enhances As tolerance by upregulating the ascorbate–glutathione cycle and glyoxalase system [148].

Conversely, there are plants that accumulate high levels of As [149]; hyperaccumulator plants reduce As (III) to As(V), and then, the inorganic As is separated into vacuoles. These plants gained a very important trait during the evolution of land plants: the ability to withstand high concentrations of As without suffering phytotoxic effects [149,150].

## 4. Transcriptomes and Metabolic and Functional Characterization of Metal-Toxicity-Related Genes

**Figure 2:**
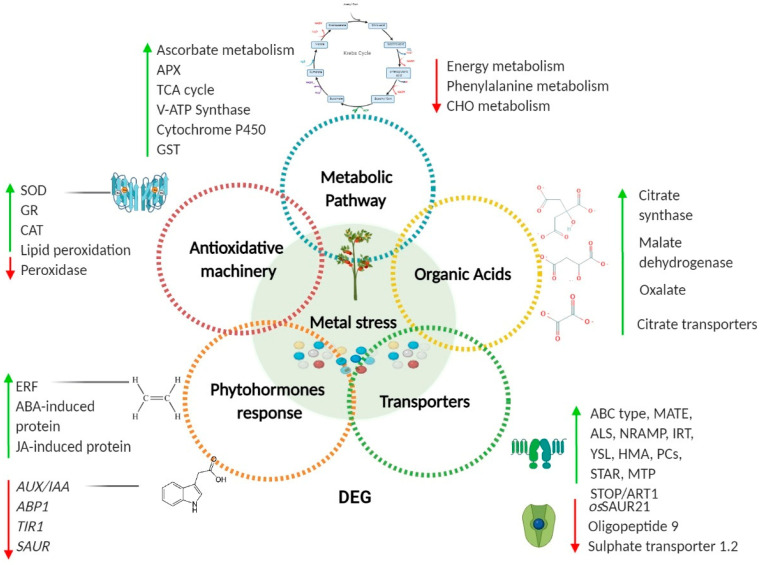
General diagram of the Gene Ontologies (GOs) of up- and downregulated genes under different metal stress conditions in plants by transcriptomic analysis. Venn diagram showing the differentially expressed genes (DEGs) up- (green arrow) and down- (red arrow) regulated that belong to the GOs of reactive oxygen species (ROS) production and antioxidative machinery, transporter-like genes (plasma membrane or vacuoles), metabolic pathways (such as the TCA cycle, which provides OAs) and phytohormonal genes in responses to As(V), Cd, Pb, and other metals. Colored circles: heavy metals, CHO: carbohydrates, TCA: tricarboxylic acid, APX: ascorbate peroxidase, ERF: Ethylene Response Factor, AUX/IAA: Auxin/Indole-3-Acetic Acid, ABP1: Auxin binding protein 1, TIR1: Transport inhibitor response 1, SAUR: small auxin upregulated RNA, OsSAUR21: auxin-responsive SAUR gene family member of *Oryza sativa*.

In recent years, transcriptomic analyses have focused on the integrated response of several metals and metalloids, which cooperate in plants under stress. The main purpose of these analyses has been to unveil the mechanisms that are shared among plants to cope with different toxic and non-toxic elements present in the soil, and to determine the form in which toxic elements take advantage of existing metal transporters and liberate chelating compounds, allowing them to enter the plant cell. Another aspect of this type of analysis is the translocation or accumulation of toxic elements within the different complex cell structures that make up plant roots. Furthermore, the use of technologies such as microarrays, RNAseq, and wide genome analysis has increased the amount of transcriptomic and genomic information available for plants under metal stress [135,151,152,153,154,155,156,157,158,159]. When analyzing the different plant transcriptomes under conditions of metal/metalloid stress, conserved trends have been observed. In fact, upregulated genes reported in different articles can be classified based on their Gene Ontology (GO) outputs in five main categories: metabolic pathways, organic acids, transporters, phytohormones, and ROS production (Figure 2). The results observed in these categories might vary according to the plant species, developmental stage, environmental conditions, the type of metal/metalloid to which the plant is been exposed, its concentration, and whether the plant is sensitive/tolerant or hyperaccumulative, among others (Table 2).

As expected, many of the differentially expressed genes (DEGs) are involved in the antioxidative machinery [152,154,167,168], transport, [151,156,163], and cell wall biosynthesis [157]; therefore, the expression (constitutive or metal stress induced) of such genes is vital for the plant, mostly in the roots during the first stages of metal stress. The localization of some transporter-like genes within the cell structure (plasma membrane or vacuoles) provides knowledge of which mechanisms are preferred by plants at the molecular level [163,164]. Among the main upregulated metabolic pathways, the upregulation of the TCA cycle is one of the common features of plant metal stress tolerance. Further, the oxidative environment provided by the alteration in mitochondrial functions (transmembrane potential impairment) causes an increase in membrane permeability, activating members of the programed cell death machinery, such as the caspase-3-like pathway [168]. The TCA cycle provides citrate and malate, which are heavy metal and metalloid chelators that are liberated by the plant as part of the first defense strategies upon contact with the toxic element [168].

The overproduction of ROS, overall mitochondrial dysfunction, and the upregulation of antioxidant enzymes such as GST, SOD, CAT, and peroxidase, among others, are common responses in plants from different phylogenic backgrounds. This indicates that the response mechanism of plants during metal toxicity or tolerance is highly conserved [152,167,169]. For example, when Al enters the mitochondria, it alters the electron transport chain, decreasing ATP production and thus increasing ROS production [170]. Additionally, enzymes that are fundamental for the TCA cycle are upregulated under metal stress; accordingly, the presence of citrate synthase, malate dehydrogenase, and malic enzyme, among others, has been reported [171,172]. On the other hand, glutathione is a molecule belonging to the low molecular thiol group that has a high affinity for toxic metals. Besides GSH, cysteine also belongs to this group. Both of these compounds are important for the production of PCs, which help to sequester and transport toxic elements into vacuoles [47].

In a study on *F. esculentum* conducted by our research group, we were able to identify, through a microarray analysis, the upregulation of TCA-cycle-related enzymes (citrate synthase, NAD-dependent malic enzyme 2) under Al-induced stress. PCS1 was also upregulated, and this corresponded to the above-described phenomenon (unpublished data). In *F. esculentum* and *F. tataricum*, a set of important transport-related genes were found to be upregulated in the first hours of Al exposure [163,164]. As expected from previous studies, *FeALS1, FeMATE1,* and *FeMATE 2* were upregulated in *F. esculentum* [163]. A comparison of these two closely-related species at the genome-wide level revealed conserved tolerance mechanisms and the upregulation of *FtFRD* genes (*FtFRDL1* and 2) and ART-Like genes (*ARL1* and 2) among other genes [164]. Recently, a transcriptomic analysis of *F. tataricum* leaves in response to Pb tolerance indicated that most of the DEGs were grouped into cell wall, binding, and energy metabolism functions. Five genes were evaluated in a yeast model, among them Metal Transporter Protein C2 (MTPC2), phytochelatin synthetase-like family protein (PCSL), a vacuolar cation/proton exchanger 1a (VCE1a), NRAMP3, and phytochelatin synthetase (PCS). All of them helped to increase the Pb tolerance in the yeast model [156] (Table 2).

The first transcriptomic research analysis in plants under conditions of metal stress or tolerance focused on identifying the main transporters (ABC transporters, NRAMPs, and STARs, among others) that help plant cells to internalize and compartmentalize toxic ions or to excrete organic acids (citrate, malate, etc.) [149,160,161]. However, interest in the role of secondary-metabolism-derived compounds is increasing, for example, commonly found DEG accessions correspond to chalcone synthase, phenylpropanoids, flavonoids, and terpenes [135,154]. Additionally, an increase in the role of ABA signaling is gaining interest; for example, in *S. orientalis* L., the mechanistic model presented after the transcriptomic analysis of this plant under conditions of Cd stress revealed that ABA could play a role in gene expression, which, in turn, activates transcription factors and response elements in the nucleus, directing the plant transcription process to the synthesis of antioxidant enzymes (GSH, CAT, or peroxidase), and at the same time, triggering phenylpropanoid biosynthesis, leading to an increase in cell wall compounds [151]. 

A similar case, which was previously discussed in this review, was reported in *C. sinensis*, where epigallocatechin gallate and polymeric proanthocyanidins were found to be part of secondary metabolites that form complexes with Al ions as part of the resistance machinery in tea plants [116].

Regarding terpene biosynthesis, this can also be deregulated because of toxic metals in the environment. A transcriptomic analysis of *F. arundinacea* revealed several unigenes that are directly related to terpene biosynthesis: monoterpenes, sesquiterpenes, diterpenes, carotenoids, brassinosteroids, and zeatins. In fact, limonene and pinene degradation related genes were found to be upregulated in the Pb-hyperaccumulative variety, whereas in the Pb-tolerant variety, the deregulation of carotenoid biosynthesis and zeatin biosynthesis related enzymes was observed [135].

Plants also respond to heavy metal or metalloid stress by increasing the levels of important plant phytohormones, such as auxins, SA, gibberellins, brassinosteroids, strigolactones, ethylene, and ABA, due mainly to their role as important plant chemical messengers for both biotic and abiotic stressors; thus, several studies have indicated that their exogenous application onto plants undergoing metal/metalloid stress has a positive effect by restoring some of the metabolic functions lost or decreased under stress. In previous years, many authors have reviewed the effects of metals/metalloids on phytohormone levels and signaling; thus, we do not go into detail in this regard. We encourage the reader to revise the related literature [173,174,175].

However, the exact mechanism of how metal/metalloid stress triggers alterations in auxin distribution, mainly in the root apices, is not fully understood. Nevertheless, investigations that suggest that metals such as Cu, Cd, and Al can exert changes to auxin distribution patterns and auxin homeostasis-related genes also are distinctly altered in plant roots under stress [160,173]. In addition, ROS interacts with the plant defense hormone SA; this has been demonstrated in *Arabidopsis* and *O. sativa*, where ABNORMAL INFLORESCENCE MERISTEM (*AIM1*) plays a key role in the SA biosynthetic pathway and is required for meristem development. SA maintains the root meristem activity by inducing the activity of WRKY transcriptional repressors, promoting ROS accumulation, which represses the expression of redox and ROS-scavenging genes and downregulates the repression of antioxidative enzymes, such as glutathione and catalases [176].

An overall increase in metal/metalloid toxic compounds above the tolerance threshold generates an immediate response in plants. Depending on the plant species and many other factors, some of them could organize the defense against these compounds or ions, avoiding as much harm as possible at both the metabolic and molecular levels. Metals such as Cu inhibit the expression of auxin biosynthetic and catabolic genes and inhibit root elongation, affecting the meristematic and elongation zones. This indicates that Cu can, in turn, modulate auxin distribution [177]. Ethylene production is also affected by metal/metalloid stress, and its presence aids in the production of antioxidant intermediaries in plant metabolism and interacting with signaling molecules. In fact, in plants, ethylene response factors constitute one of the gene families that are differentially expressed after metal stress [178] (Table 2). 

High-throughput sequence analysis with metal accumulator and tolerant plants has demonstrated the up- and downregulation of several microRNAs (miRNAs) in plants exposed to metals (Zn, Cd, and Cu). MiRNAs trigger a cascade of signals that facilitate genome stress related metal responses with substantial roles in supplementing metal detoxification regulating transcription factors (TF), undergoing gene regulation by miRNA targets (such as ABC or NRAMP transporters) with direct functions in metal tolerance pathways, and generating tools for the early monitoring of plant environmental stressors [179,180,181].

The interesting results that can be obtained by means of transcriptomics, wide genome association mapping, and more recently, metabolomics, phenomics, and ionomics will help us to understand how plants prepare themselves and change under different environmental conditions, which is a fascinating example of the plants’ genomes, transcriptomes, and metabolome plasticity. This contributes to the understanding of metal/metalloid stress in plants and the mechanisms involved at the genetic level. Further, these analyses can be utilized to discover the behavior of hyperaccumulative plants, which can be employed for biotechnological purposes with the goal of phytoremediation to reduce, in part, the increasing metal and metalloid concentrations in soil around the globe.

## 5. Biotechnological and Ecological Applications of Accumulator and Hyperaccumulator Plants

The analysis of plants with the ability to accumulate and hyperaccumulate has increased in recent years [34]. Furthermore, biomolecular tools are been utilized to rapidly identify plants, pathways, and even genes related to metal accumulation. Genetic transformation techniques help to obtain plants that exhibit heavy metal resistance by manipulating metabolic pathways responsible for stress defense reactions. Furthermore, they allow us to identify biochemical and molecular alterations caused in response to metal stress in plants and the genes, specific proteins, and transporters involved in the detoxification of toxic metals in plants [63,182].

Molecular markers can be used to map quantitative trait loci (QTLs) or amplify fragment length polymorphisms (AFLPs), for hybrid development, and as aids in searching for genes and pathways within wild plant relatives. They could be introduced onto commercial crops to render hyperaccumulative traits. The identification of fast growing and better metal accumulating plant genotypes can also be used to obtain specific traits by overexpressing genes related to DNA repair and transcription, antioxidant machinery, chelators, and chloroplast genes [182,183]. MicroRNAs (miRNAs) regulate gene expression in both plants and mammalian systems under conditions of biotic or abiotic stress.

Gene editing technologies, such as customized homing nuclease (meganuclease), zinc-finger nucleases (ZNFs), transcription activator-like effector nucleases (TALENs), and CRISPR-Cas9 (clustered regularly interspaced short palindromic repeats (CRISPR)-associated protein 9), have been successfully used for genome editing in bacteria, mammals, and plants [107,184]. The CRISPR/Cas9 technique could be used to introduce mutagenesis in sequences of metal transporter genes to enhance their activity or modify their selectivity and could also enhance the ability of plants to uptake metals and translocate in shoots. This is a promising tool to obtain yield-competitive, low-metal accumulated plants [185]. All of these approaches and techniques might be useful for the development of more tolerant transgenic plants for environmental cleanup and restoration of the soil quality through phytoremediation (Table 3).

## 6. Conclusions and Future Prospects

Modifications to the availability and distribution of metalloids that function as plant nutrients or the presence of toxic, nonbiological metals in soil induce various adaptive mechanisms in plants (uptake and exclusion transporters, OAs, enzymatic and oxidative responses, and chelators), highlighting the importance of these molecules within plants in response to abiotic stress. Through the examples reviewed here, we inferred a high degree of specialization among plants. Such capacity must certainly have been obtained through evolution; however, more recently domesticated crops must face rapid adaptation to the growing areas of contaminated soils. Nevertheless, as has occurred with noncrop plants, studies have confirmed their capability to utilize these essential elements (as transporters and translocation machinery) to diminish metal/metalloid stress. A common trend amongst all the given examples is the use of pre-existing transporters by the plant to introduce toxic compounds into sites of accumulation inside the plant cell. Such a phenomenon takes place on the natural barrier between the plant cell and the rhizosphere: the plant cell wall. Changes could occur in this cell barrier under conditions of metal/metalloid toxicity. The prospective use of these technologies in the environment can help to ameliorate the rise of soil pollution in different areas around the world. Therefore, it is vital to continue this type of applied research using the scientific basis provided by both physiological and omic approaches to propose a solution to the decrease of arable land in vast areas of our planet due to soil acidification and contamination.

The knowledge gathered from these findings can help to increase the production and yield of staple crops such as maize, rice, and wheat. Additionally, the use of alternative cycles between economically important agronomic crops and those with phytoremediation capacity should be used. Biotechnological tools, such as the use of byproducts and transgenic crops for phytoremediation, are now considered important strategies to overcome soil pollution and meet the demands for arable land worldwide for the next generations.

## Figures and Tables

**Table 1:** Toxic effects of metals and metalloids in different plant systems.

Metal	Plant Model	Entrance	Chelation/Translocation	Accumulation	Effects	References
As	*Oryza sativa* *Arabidopsis thaliana*	Aquaporins [(As(III)] Roots P transporters [As(V)]	AtNIP5;1, AtNIP6;1, AtNIP7, OsNIP2;1, OsNIP3;2, AtABCCJ AtABCC2	Roots Shoots, Leaves, not always available in edible parts. PCS	ROS increase Lipid peroxidation	[61,62] [63,64]
Zn	*Arabidopsis thaliana* *Zea mays*	Zn transporters Roots ZRT/IRT YS YSL	PCS+GSH MTs Citrate, malate At-PCR2 root to shoot translocation AtMPT1 translocation to vacuoles AtHMA2-AtHMA4 Fe-phytosiderophores	Tonoplast, vacuoles from dividing cells and roots (YS) Leaves to seeds during senescence (YSL)	Photosynthesis and growth inhibition Chlorosis ROS increase	[65,66,67,68]
Cd	*Arabidopsis thaliana*	NI *	AtABCC1 and AtABCC2 GSH Cd(II) transport AtABCC3	NI	Growth inhibition, nutrition imbalance, photosynthesis supression, chlorosis, ROS increase	[69,70]
Al	*Arabidopsis thaliana*	Root system Cell wall Plasma membrane Symplasm Apoplasm	ALMT (malate), MATE (citrate), STAR1-STAR2. ALS3-ALS1 FeALS3 AvABCG1 Oxalate	Cell wall Leaf Roots Tonoplast Vacuoles	Growth inhibition ROS increase Lipid peroxidation	[71,72,73,74]
Cu	*Theobroma* *cacao* *Arabidopsis thaliana* *Oryza sativa*	NI *	GSH. PCs, MTs, phytochelatins, YSL, COP, Cu transporters, ERF	Roots Leaves Stem	ROS increase, lipid peroxidation, ionic leakage, protein and nucleic acids damage, changes in chloroplasts, thylakoids. Plasmolysis, chlorosis, rolling of leaves	[47,75,76,77,78]
Pb	*Lactuca sativa* *Triticum aestivum* *Tetraena qataranse*	H+/ATPase systems Apoplasm	Thiol compounds PCs, MTs	Inactivation in vacuoles	Stimulation of ROS Disrupts the electron transport in the energy transformation efficiency of photosystem II	[79,80,81,82]

* NI: Not identified.

**Table 2:** Transcriptomic studies in plants under conditions of metal and metalloid stress.

Plant Model	Metal/Metalloid	Differentially Expressed Upregulated Genes	Reference
*Platanus acerifolia*	Pb	Photosynthesis, gibberellins, glutathione, antioxidant enzymes chelating compounds Metal transporters Detoxification mechanisms	[160]
*Zea mays*	Pb	ZmNAS2, ZmNAS4, ZmNAS9, transcription factors, cell wall synthesis, metal redox, ethylene response factors	[161]
*Oryza sativa*	Cd, As, Pb, Cr	Secondary metabolites, flavonoid biosynthesis, lipid metabolism, AA metabolism, CHO metabolism, xenobiotic biodegradation, ascorbate and alderate metabolism, membrane transport, multidrug resistance proteins, iron coupled transporters, major facilitator superfamily, ABC transporters, GST, MAPK signaling pathway proteins.	[149]
*Oryza sativa*	Cr(VI)	Binding activity, metabolic and cellular process proteins, catalytic activity, ethylene related, cytokinin, MAPK pathway, CDKs, ubiquitin proteasome.	[162]
*Zea mays*	Al	TCA cycle, auxin related genes, gibberellic (GA) and jasmonic acid (JA) pathway, brassinosterioids pathway	[74]
*Oryza sativa (Fe deficient)*	Cd	Heavy metal transporters, auxin, NO and gibberellic acid pathway proteins, YSL genes	[149]
*Fagopyrum esculentum Moench cv Jianxi*	Al	STOP/ART1, FeSTAR1, FeALS3, FeALS1, FeMate, FeMate2	[163]
*Fagopyrum tataricum*	Al	Citrate transporters, ART1/STOP, ALS1, STAR1, STAR2/ALS3, antioxidant activity, xenobiotics biodegradation, lipid metabolism, FtFRDL1, FtFRDL2	[164]
*Populus x canadensis*	Zn	Redox process, transport and cellular Fe ion homeostasis, ZIP2, NRAMP1, metal chelation, detoxification or glutathione conjugated molecules, metal uptake	[165]
*Hordeum vulgare* (Cd sensitive and Cd tolerant varieties)	Cd	CHO metabolism, catalase, ABA induced protein, JA induced protein 1, defensin, chitinase, dehydrin, ABC transporters, β- ketoacyl CoA synthase, acyl CoA synthase, cytochrome p450, V-ATP synthase, expansin, β-xylosidase	[166]
*Medicago sativa*	Pb	GO enriched pathways: binding, transport, membranes, signaling and energy metabolism. DEGs for POD, SOD, GST, flavonoids and isoflavonoids, chalcone synthase, ABC transporters, IRT, CDFs, WRKY, ERFs and bZIP	[151]
*Festuca arundinacea*	Pb	GO enrchiched in the metabolisms for energy production, terpenoids, poliketides and carbohydrates. Zeatin biosynthesis was increased as well as limonene and pinene degradation,	[135]
*Oryza sativa* L. indica	Cd, As	Redox control, stress response, transcriptional regulation, transmembrane transport, signal transduction, macromolecule and sulfur compound metabolism	[152]
*Cunninghamia lanceolata* (Lamb.) Hook	Al	Cell wall and lipid metabolism, signaling pathways and secondary metabolism, flavonoids and phenylpropanoids. Transcription factors such as bHLH, C2H2, ERF, bZIP, GRAS and MYB	[155]
*Verbena* *bonariensis*	Cd	Lignin synthesis, chalcone synthase (CHS), anthocyanidin synthase (ANS)	[153]
*Siegesbeckia orientalis*	Cd	NRAMP5 isoform X1, HMA genes, ABC transporter 1, Pleotropic Resitance 1 and 8, GSH, CAT, Peroxidase, L-ascorbate peroxidase, phenylpropanoid biosynthesis	[154]
*Gossypium hirsutum*	Cd	Oxidation-reduction process, metal ion binding. DEGs for metal transporter genes ABC, CDF, HMA, annexin genes and heat shock proteins. *GhHMAD5* aids in Cd tolerance	[167]
*Fagopyrum tataricum*	Pb	Metal Transporter Protein C2 (MTPC2), phytochelatin synthetase-like family protein (PCSL), a Vacuolar cation/proton exchanger 1a (VCE1a), NRAMP3, and phytochelatin synthetase (PCS),d	[156]
*Glycine max (2 varieties: M90-24 and Pella)*	Al	11 genes enriched in the GO for cellulose production: cellulose synthases, which indicates an important role for cellulose in soybean Al tolerance	[137,157]

**Table 3:** Biotechnological strategies for heavy metal tolerance, accumulation, and phytoremediation in plants.

Plant Model	Toxic Metal	Strategy Employed	Main Results	References
*Medicago sativa*	Trichloroethylene (TCE) contaminants	Genetic transformation	Transgenic alfalfa plants coexpressing GST and human P4502El (CYP2El) resulted in an increased resistance and accumulation of heavy metals.	[186]
*Nicotiana tabacum*	PBC Delor 103	Genetic transformation	*bphC,* a bacterial gene from the degradation pathway of polychlorinated biphenyls (PBS). The *bphC* codes for an enzyme 23-dihidroxybipbenyl-1,2-dioxygenase. Production of higher biomass.	[187]
*Brassica juncea*	Ni	Genotype screening in hydroponically grown plants	10 genotypes screened in hydroponic culture with varying concentrations of nickel chloride (0–50 µM). One genotype, Pusajai Kisan was the most tolerant accumulating up to 1.7 µg Ni g-1 dry weight in aerial parts	[188]
*Nicotiana tabacum*	Hg	Genetic transformation	Two transgenic lines were transformed with *merT*, *merP*, *merBJ*, and *merB2* from Hg resistant *Pseudomonas* K-62. PpK polyphosphate kinase from *Enterobacter aerogenes. PCSI*, phytochelatin syntbase from *N. tabacum.* Both transgenic lines were more resistant to organic Hg against the WT. In the Ntp-36 roots, Hg content was up to 251 µg/g.	[189]
*Oryza sativa*	Cd	Knockdown *OsNRAMP5* by RNAi	Cd phytoremediation was done producing *OsNRA.MP5* RNAi plants using the natural high Cd accumulating cultivar Anjana Dhan (A5i). The biomass of A5i was unchanged by suppression of *OsNRA.MP5* expression. These plants accumulated twice as Cd in their shoots as the WT plant.	[190]
*Festuca arundinacea*	Cd	Genetic transformation	*PaPCSJ* phytochelating synthase 1; *PaGCS* alpha- glutamyl cysteine synthetase gene. Single and double transformants showed increase tolerance and accumulation of Cd and PC than WT plants. PaGCS appears to have greater influence that *PaPCS* over synthesis and Cd tolerance/accumulation.	[191]
*Beta vulgaris*	Cd, Zn, Cu, or combinated	Genetic transformation	y-glutamylcysteine synthetase-glutathione synthetase (StGCS-GS) from *Streptococcus thermophilus.* This GCS has limited feedback inhibition and was overexpressed in sugar beet. Transgenic sugar beets accumulated more Cd, Zn, and Cu ions in shoots than WT and higher GSH and PC levels under different metal stresses. When multiple metals were present at the same time transgenic sugar beets resisted 2 or 3 metal concentrations at the same time.	[192]
*Arabidopsis thaliana*	Al	Genetic transformation	*SbGlu*l, which encodes a β*-1,3-*glucanase enzyme, was expressed in *A. thaliana.* Greater Al tolerance than WT plants was observed due to an increase in a P-1,3-glucanase with decrease in Al accumulation and a decrease in callose deposition.	[193]
*Arabidospis thaliana*	Al	Genetic transformation	*GmARI* gene codes for soybean Ariadne-like ubiquitin ligase gene. Overexpressed in *A.* *thaliana* enhanced Al tolerance.	[194]
*Fagopyrum. esculentum*	Cr(VI)	Removal of Cr(VI) from aqueous solutions	pH had significant effect on Cr(VI) removal; optimal pH = 2.0. Removal was analyzed using batch experiments. Cr(VI) was partially reduced to Cr(III). The proposed mechanisms for Cr(VI) removal using buckwheat hull were found by electrostatic attraction, chemical reduction and complex interaction.	[195]
*Helianthus annus*	Cu	Cu-phytoaccumulation	Plants grown in the two vineyard soils showed an increase in height. The bioaccumulation factors (BFC) decreased in plants grown in the three different types of soils, suggesting that they were able to accumulate Cu within them.	[196]
*Arabidopsis thaliana*	Cr(VI)	Analysis of two transgenic lines	*At.PTJ* and *AtPT2* genes in *A. thaliana* transgenic lines When exposed to 140 µM potassium chromate, an increase in levels of Pi and sulfate was observed and a supplementation to the seedlings with Cr(VI) toxicity completely and partially restored the root growth, respectively.	[197]
*Brassica napus*	Cu	Analysis of EDTA effect of phytoextraction	Cu alone significantly decreased plant growth biomass, photosynthetic pigments, and gas exchange characteristics. Cu stress also reduced the activities of antioxidants such as SOD. POD, APX, and CAT. The application of EDTA significantly alleviated Cu-induced toxic effects.	[198]

## Data Availability

Not applicable.
